# 2-(3,5-Dimethyl-1,1-dioxo-2*H*-1λ^6^,2,6-thia­diazin-4-yl)benzoic acid

**DOI:** 10.1107/S1600536812037026

**Published:** 2012-09-05

**Authors:** Nilay Bhatt, Pralav Bhatt, Thavendran Govender, Hendrik G. Kruger, Glenn E. M. Maguire

**Affiliations:** a Chemistry Department, JJT University, Rajasthan, India; b School of Chemistry, University of KwaZulu-Natal, Durban 4000, South Africa; c School of Pharmacology, University of KwaZulu Natal, Westville Campus, Private Bag X54001, South Africa

## Abstract

In the title mol­ecule, C_12_H_12_N_2_O_4_S, the S atom of the thia­diazine ring deviates by 0.5104 (4) Å from the mean plane of the other five atoms [largest deviation = 0.0623 (15) Å] giving a slightly distorted sofa conformation. The carb­oxy H atom was refined as disordered over two sets of sites with refined occupancies of 0.58 (2) and 0.48 (2). This corresponds to rotational disorder of the C=O and O—H groups about the attached C—C bond. In the crystal, O—H⋯O and N—H⋯O hydrogen bonds connect the mol­ecules into chains along [110].

## Related literature

The title compound is a phenyl acid thia­diazine derivative. For synthetic background and applications of 1,2,6-thia­diazine-1,1-dioxide derivatives, see: Wright (1964 ▶); Breining *et al.* (1995 ▶). For a related structure, see: Bhatt *et al.* (2012 ▶)
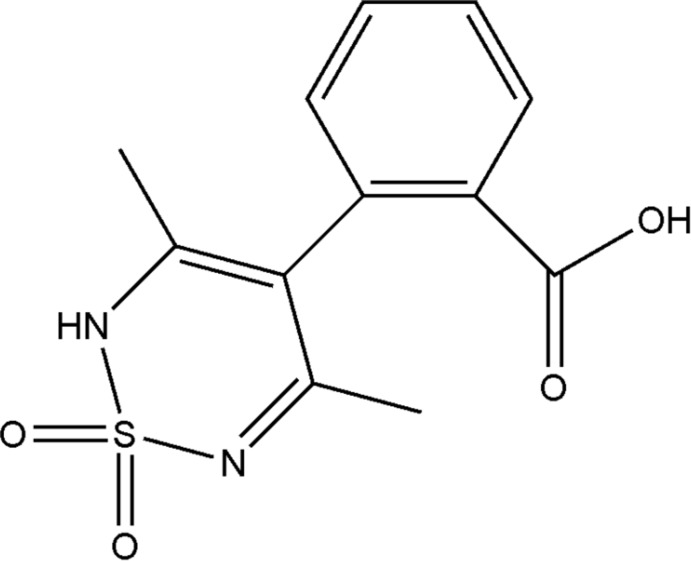


## Experimental

### 

#### Crystal data

C_12_H_12_N_2_O_4_S
*M* *_r_* = 280.30
Monoclinic, 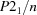

*a* = 10.5048 (14) Å
*b* = 10.4254 (13) Å
*c* = 11.1294 (14) Å
β = 92.772 (4)°
*V* = 1217.4 (3) Å^3^
*Z* = 4
Mo *K*α radiation
μ = 0.28 mm^−1^
*T* = 173 K
0.24 × 0.19 × 0.18 mm

#### Data collection

Bruker Kappa DUO APEXII diffractometer
Absorption correction: multi-scan (*SADABS*; Bruker, 2006 ▶) *T*_min_ = 0.936, *T*_max_ = 0.952
5724 measured reflections
3030 independent reflections
2573 reflections with *I* > 2σ(*I*)
*R*_int_ = 0.020

#### Refinement

*R*[*F* ^2^ > 2σ(*F* ^2^)] = 0.038
*wR*(*F* ^2^) = 0.103
*S* = 1.05
3030 reflections
187 parameters
3 restraints
H atoms treated by a mixture of independent and constrained refinement
Δρ_max_ = 0.37 e Å^−3^
Δρ_min_ = −0.42 e Å^−3^

### 

Data collection: *APEX2* (Bruker, 2006 ▶); cell refinement: *SAINT* (Bruker, 2006 ▶); data reduction: *SAINT*; program(s) used to solve structure: *SHELXS97* (Sheldrick, 2008 ▶); program(s) used to refine structure: *SHELXL97* (Sheldrick, 2008 ▶); molecular graphics: *OLEX2* (Dolomanov *et al.*, 2009 ▶); software used to prepare material for publication: *SHELXL97*.

## Supplementary Material

Crystal structure: contains datablock(s) I, global. DOI: 10.1107/S1600536812037026/lh5507sup1.cif

Structure factors: contains datablock(s) I. DOI: 10.1107/S1600536812037026/lh5507Isup2.hkl

Supplementary material file. DOI: 10.1107/S1600536812037026/lh5507Isup3.cml

Additional supplementary materials:  crystallographic information; 3D view; checkCIF report

## Figures and Tables

**Table 1:** Hydrogen-bond geometry (Å, °)

*D*—H⋯*A*	*D*—H	H⋯*A*	*D*⋯*A*	*D*—H⋯*A*
N1—H1⋯O1^i^	0.97 (1)	2.09 (2)	2.9699 (18)	151 (2)
O4—H4⋯O3^ii^	0.97 (3)	1.64 (3)	2.6103 (19)	177 (3)
O3—H3⋯O4^ii^	0.97 (3)	1.67 (3)	2.6103 (19)	161 (5)

Symmetry codes: (i) 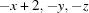; (ii) 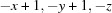.

**Fig. 1.**
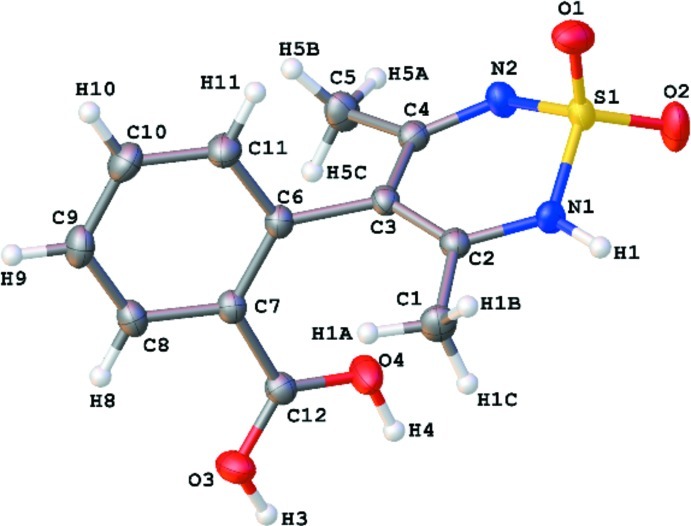
The molecular structure of the title compound with displacement ellipsoids drawn at 40% probability. Atoms H3 and H4 are disorder components.

**Fig. 2.**
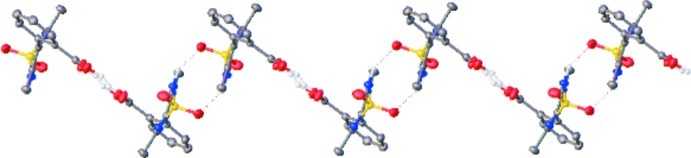
The hydrogen bonding interactions of the title compound along [110]. All H atoms except those involved in hydrogen bonding have been omitted for clarity.

## supplementary crystallographic information

### Comment

The synthesis of 1,2,6-thiadiazine-1,1-dioxides derivatives was first reported using sulfamide with alpha and beta diketones (Wright, 1964). Anti-HIV-1 activity for this family of structures was also reported (Breining *et al.*, 1995). For this reason we are interested in this class of compounds as potential agents in other diseases. The crystal structure of the title compound is described herein.

The molecular structure of the title compound is shown in Fig. 1. It is the second 3,5-dimethyl based structure reported with an aromatic ring at position 4 of the thiadiazine ring. Previously we have reported the phenyl ethyl and methyl ester (Bhatt *et al.*, 2012). It is the first containing an acid functional group in the broader family of 1,2,6-thiadiazine-1,1-dioxides. The S atom of the thiadiazine ring deviates by 0.5104 (4) Å from the plane of the other five atoms [largest deviation 0.0623 (15) Å] giving a slightly distorted sofa conformation. The carboxylic acid H atom was refined as disordered over two sets of sites with refined occupancies 0.58 (2) and 0.48 (2). This corresponds to roational disorder of the C═O and O—H groups about the attached C—C bond. In the crystal, O—H···O and N—H···O hydrogen bonds connect molecules into chains along [110] (Fig. 2).

### Experimental

2-(2, 4-dioxopentan-3-yl) benzoic acid (0.072 mol) and sulfamide (0.072 mol) were dissolved in methanol (70 ml). Anhydrous hydrogen chloride gas was bubbled into the mixture until the temperature increased to 323 K. The contents of the reaction were then refluxed for 3hrs. The reaction mixture was cooled, filtered and the filtrate was concentrated under reduced pressure. The residual solid was treated with NaOH (0.138 mol) in water (200 ml), the contents were heated at 343 K for 2.5 hrs. The reaction progress was monitored by TLC ethyl acetate/hexane (80:20 *R*_f_ = 1/2). The reaction mixture was cooled and acidified using concentrated HCl to get the crude acid as an oil. To this oily residue was added a solution of methanol/ethyl acetate (10 ml) (10/90) which yielded a white colourless solid (79%). *M*.p.= 523 K. Crystals suitable for X-ray analysis were grown in dioxane/water at room temprature.

### Refinement

All hydrogen atoms, except H1, H3 and H4, were placed in idealized positions and refined with geometric constraints [C—H = 0.95 - 0.98 Å and *U*_iso_(H) = 1.2*U*_eq_(C) or 1.5*U*_eq_(C_methyl_). The hydrogen atom H1 was located in a difference Fourier map and refined with O—H distance restraint to the value of 0.97 (1) Å. The carboxy hydroxyl hydrogen is distributed over two sites: H3 and H4, were both located in a difference Fourier map and refined with a O—H distance restraint to the value of 0.97 (1) Å. The site occupancy factors refined to 0.48 (8) for H3 and 0.52 (8) for H4.

### Crystal data

**Table:**

C_12_H_12_N_2_O_4_S	*F*(000) = 584
*M**_r_* = 280.30	*D*_x_ = 1.529 Mg m^−^^3^*D*_m_ = 0 Mg m^−^^3^*D*_m_ measured by not measured
Monoclinic, *P*2_1_/*n*	Melting point: 523 K
Hall symbol: -P 2yn	Mo *K*α radiation, λ = 0.71073 Å
*a* = 10.5048 (14) Å	Cell parameters from 5724 reflections
*b* = 10.4254 (13) Å	θ = 2.6–28.4°
*c* = 11.1294 (14) Å	µ = 0.28 mm^−^^1^
β = 92.772 (4)°	*T* = 173 K
*V* = 1217.4 (3) Å^3^	0, colourless
*Z* = 4	0.24 × 0.19 × 0.18 mm

### Data collection

**Table:**

Bruker Kappa DUO APEXII diffractometer	3030 independent reflections
Radiation source: fine-focus sealed tube	2573 reflections with *I* > 2σ(*I*)
Graphite monochromator	*R*_int_ = 0.020
0.5° φ scans and ω scans	θ_max_ = 28.4°, θ_min_ = 2.6°
Absorption correction: multi-scan (*SADABS*; Bruker, 2006)	*h* = −12→14
*T*_min_ = 0.936, *T*_max_ = 0.952	*k* = −13→12
5724 measured reflections	*l* = −11→14

### Refinement

**Table:**

Refinement on *F*^2^	Primary atom site location: structure-invariant direct methods
Least-squares matrix: full	Secondary atom site location: difference Fourier map
*R*[*F*^2^ > 2σ(*F*^2^)] = 0.038	Hydrogen site location: inferred from neighbouring sites
*wR*(*F*^2^) = 0.103	H atoms treated by a mixture of independent and constrained refinement
*S* = 1.05	*w* = 1/[σ^2^(*F*_o_^2^) + (0.0518*P*)^2^ + 0.4988*P*] where *P* = (*F*_o_^2^ + 2*F*_c_^2^)/3
3030 reflections	(Δ/σ)_max_ = 0.001
187 parameters	Δρ_max_ = 0.37 e Å^−^^3^
3 restraints	Δρ_min_ = −0.42 e Å^−^^3^

### Special details

**Table:**

Geometry. All e.s.d.'s (except the e.s.d. in the dihedral angle between two l.s. planes) are estimated using the full covariance matrix. The cell e.s.d.'s are taken into account individually in the estimation of e.s.d.'s in distances, angles and torsion angles; correlations between e.s.d.'s in cell parameters are only used when they are defined by crystal symmetry. An approximate (isotropic) treatment of cell e.s.d.'s is used for estimating e.s.d.'s involving l.s. planes.
Refinement. Refinement of *F*^2^ against ALL reflections. The weighted *R*-factor *wR* and goodness of fit *S* are based on *F*^2^, conventional *R*-factors *R* are based on *F*, with *F* set to zero for negative *F*^2^. The threshold expression of *F*^2^ > σ(*F*^2^) is used only for calculating *R*-factors(gt) *etc*. and is not relevant to the choice of reflections for refinement. *R*-factors based on *F*^2^ are statistically about twice as large as those based on *F*, and *R*- factors based on ALL data will be even larger.

### Fractional atomic coordinates and isotropic or equivalent isotropic displacement parameters (Å^2^)

**Table:**

	*x*	*y*	*z*	*U*_iso_*/*U*_eq_	Occ. (<1)
S1	0.98957 (3)	0.13712 (4)	0.12970 (3)	0.02250 (12)	
O1	1.01274 (12)	0.00736 (13)	0.16838 (11)	0.0344 (3)	
O2	1.09339 (11)	0.20118 (15)	0.07866 (12)	0.0395 (3)	
O3	0.39308 (11)	0.41036 (12)	0.06653 (11)	0.0311 (3)	
O4	0.60472 (11)	0.39428 (13)	0.08005 (13)	0.0353 (3)	
N1	0.87045 (12)	0.13786 (13)	0.02772 (11)	0.0231 (3)	
N2	0.93605 (12)	0.21970 (13)	0.23740 (12)	0.0241 (3)	
C1	0.65248 (16)	0.09799 (18)	−0.03955 (14)	0.0285 (3)	
H1A	0.5677	0.0883	−0.0076	0.043*	
H1B	0.6784	0.0168	−0.0754	0.043*	
H1C	0.6500	0.1653	−0.1010	0.043*	
C2	0.74610 (14)	0.13394 (14)	0.06012 (13)	0.0201 (3)	
C3	0.71616 (13)	0.16832 (14)	0.17403 (13)	0.0192 (3)	
C4	0.81280 (14)	0.22158 (14)	0.25478 (13)	0.0208 (3)	
C5	0.77422 (16)	0.29238 (17)	0.36412 (15)	0.0293 (4)	
H5A	0.8483	0.3374	0.4007	0.044*	
H5B	0.7416	0.2314	0.4222	0.044*	
H5C	0.7076	0.3547	0.3412	0.044*	
C6	0.58294 (14)	0.15746 (14)	0.21603 (13)	0.0204 (3)	
C7	0.48160 (14)	0.24135 (14)	0.18462 (13)	0.0206 (3)	
C8	0.36101 (15)	0.21905 (16)	0.22911 (14)	0.0252 (3)	
H8	0.2925	0.2749	0.2067	0.030*	
C9	0.34023 (16)	0.11711 (17)	0.30514 (15)	0.0292 (4)	
H9	0.2581	0.1035	0.3349	0.035*	
C10	0.43933 (16)	0.03505 (17)	0.33774 (15)	0.0296 (4)	
H10	0.4257	−0.0349	0.3903	0.036*	
C11	0.55916 (16)	0.05546 (15)	0.29322 (14)	0.0263 (3)	
H11	0.6267	−0.0015	0.3159	0.032*	
C12	0.49362 (14)	0.35576 (14)	0.10521 (14)	0.0217 (3)	
H4	0.606 (4)	0.469 (2)	0.028 (3)	0.030 (16)*	0.52 (8)
H3	0.406 (6)	0.491 (3)	0.025 (5)	0.06 (2)*	0.48 (8)
H1	0.892 (2)	0.113 (2)	−0.0528 (8)	0.047 (6)*	

### Atomic displacement parameters (Å^2^)

**Table:**

	*U*^11^	*U*^22^	*U*^33^	*U*^12^	*U*^13^	*U*^23^
S1	0.01708 (18)	0.0281 (2)	0.02257 (19)	0.00231 (14)	0.00382 (13)	−0.00124 (14)
O1	0.0401 (7)	0.0339 (7)	0.0292 (6)	0.0161 (6)	0.0017 (5)	0.0019 (5)
O2	0.0237 (6)	0.0582 (9)	0.0374 (7)	−0.0106 (6)	0.0105 (5)	−0.0040 (6)
O3	0.0259 (6)	0.0294 (6)	0.0376 (7)	0.0023 (5)	−0.0030 (5)	0.0086 (5)
O4	0.0255 (6)	0.0308 (6)	0.0503 (8)	0.0038 (5)	0.0102 (5)	0.0165 (6)
N1	0.0207 (6)	0.0306 (7)	0.0182 (6)	0.0028 (5)	0.0029 (5)	−0.0019 (5)
N2	0.0201 (6)	0.0278 (7)	0.0246 (6)	0.0012 (5)	0.0017 (5)	−0.0048 (5)
C1	0.0275 (8)	0.0347 (9)	0.0231 (7)	−0.0019 (7)	−0.0012 (6)	−0.0041 (7)
C2	0.0201 (7)	0.0183 (7)	0.0219 (7)	0.0013 (5)	0.0018 (5)	0.0012 (6)
C3	0.0182 (6)	0.0178 (6)	0.0218 (7)	0.0022 (5)	0.0030 (5)	0.0010 (5)
C4	0.0228 (7)	0.0193 (7)	0.0204 (6)	0.0026 (6)	0.0026 (5)	0.0000 (5)
C5	0.0275 (8)	0.0350 (9)	0.0256 (7)	0.0028 (7)	0.0024 (6)	−0.0096 (7)
C6	0.0196 (7)	0.0211 (7)	0.0207 (6)	−0.0005 (6)	0.0035 (5)	−0.0006 (5)
C7	0.0191 (7)	0.0206 (7)	0.0222 (7)	−0.0010 (6)	0.0027 (5)	−0.0011 (6)
C8	0.0191 (7)	0.0281 (8)	0.0285 (7)	0.0005 (6)	0.0031 (6)	−0.0019 (6)
C9	0.0242 (8)	0.0326 (9)	0.0313 (8)	−0.0066 (7)	0.0081 (6)	−0.0011 (7)
C10	0.0338 (9)	0.0278 (8)	0.0279 (8)	−0.0048 (7)	0.0071 (6)	0.0039 (7)
C11	0.0277 (8)	0.0233 (8)	0.0281 (7)	0.0024 (6)	0.0038 (6)	0.0036 (6)
C12	0.0212 (7)	0.0202 (7)	0.0236 (7)	0.0012 (6)	0.0016 (5)	−0.0014 (6)

### Geometric parameters (Å, º)

**Table:**

S1—O2	1.4206 (12)	C3—C6	1.5011 (19)
S1—O1	1.4368 (13)	C4—C5	1.496 (2)
S1—N2	1.5998 (13)	C5—H5A	0.9800
S1—N1	1.6483 (13)	C5—H5B	0.9800
O3—C12	1.2573 (19)	C5—H5C	0.9800
O3—H3	0.9699 (10)	C6—C11	1.397 (2)
O4—C12	1.2781 (18)	C6—C7	1.409 (2)
O4—H4	0.9699 (10)	C7—C8	1.402 (2)
N1—C2	1.3724 (19)	C7—C12	1.493 (2)
N1—H1	0.9698 (10)	C8—C9	1.382 (2)
N2—C4	1.3185 (19)	C8—H8	0.9500
C1—C2	1.494 (2)	C9—C10	1.382 (2)
C1—H1A	0.9800	C9—H9	0.9500
C1—H1B	0.9800	C10—C11	1.391 (2)
C1—H1C	0.9800	C10—H10	0.9500
C2—C3	1.369 (2)	C11—H11	0.9500
C3—C4	1.435 (2)		
			
O2—S1—O1	116.12 (8)	C4—C5—H5A	109.5
O2—S1—N2	110.55 (8)	C4—C5—H5B	109.5
O1—S1—N2	110.05 (7)	H5A—C5—H5B	109.5
O2—S1—N1	107.12 (8)	C4—C5—H5C	109.5
O1—S1—N1	108.82 (8)	H5A—C5—H5C	109.5
N2—S1—N1	103.34 (7)	H5B—C5—H5C	109.5
C12—O3—H3	115 (4)	C11—C6—C7	118.02 (13)
C12—O4—H4	115 (2)	C11—C6—C3	116.49 (13)
C2—N1—S1	121.29 (10)	C7—C6—C3	125.49 (13)
C2—N1—H1	120.0 (14)	C8—C7—C6	119.63 (14)
S1—N1—H1	115.7 (14)	C8—C7—C12	116.44 (13)
C4—N2—S1	120.07 (11)	C6—C7—C12	123.94 (13)
C2—C1—H1A	109.5	C9—C8—C7	121.06 (15)
C2—C1—H1B	109.5	C9—C8—H8	119.5
H1A—C1—H1B	109.5	C7—C8—H8	119.5
C2—C1—H1C	109.5	C10—C9—C8	119.82 (15)
H1A—C1—H1C	109.5	C10—C9—H9	120.1
H1B—C1—H1C	109.5	C8—C9—H9	120.1
C3—C2—N1	119.99 (14)	C9—C10—C11	119.65 (15)
C3—C2—C1	125.51 (14)	C9—C10—H10	120.2
N1—C2—C1	114.40 (13)	C11—C10—H10	120.2
C2—C3—C4	119.62 (13)	C10—C11—C6	121.82 (15)
C2—C3—C6	121.88 (13)	C10—C11—H11	119.1
C4—C3—C6	118.44 (12)	C6—C11—H11	119.1
N2—C4—C3	124.92 (13)	O3—C12—O4	122.93 (14)
N2—C4—C5	115.67 (13)	O3—C12—C7	118.07 (13)
C3—C4—C5	119.29 (13)	O4—C12—C7	119.00 (13)
			
O2—S1—N1—C2	−152.60 (12)	C4—C3—C6—C11	−78.20 (18)
O1—S1—N1—C2	81.12 (13)	C2—C3—C6—C7	−75.8 (2)
N2—S1—N1—C2	−35.82 (14)	C4—C3—C6—C7	101.44 (18)
O2—S1—N2—C4	143.83 (13)	C11—C6—C7—C8	−1.2 (2)
O1—S1—N2—C4	−86.58 (14)	C3—C6—C7—C8	179.14 (14)
N1—S1—N2—C4	29.49 (14)	C11—C6—C7—C12	178.69 (14)
S1—N1—C2—C3	20.0 (2)	C3—C6—C7—C12	−0.9 (2)
S1—N1—C2—C1	−163.46 (12)	C6—C7—C8—C9	1.1 (2)
N1—C2—C3—C4	6.8 (2)	C12—C7—C8—C9	−178.81 (15)
C1—C2—C3—C4	−169.34 (15)	C7—C8—C9—C10	−0.3 (3)
N1—C2—C3—C6	−175.96 (13)	C8—C9—C10—C11	−0.3 (3)
C1—C2—C3—C6	7.9 (2)	C9—C10—C11—C6	0.2 (3)
S1—N2—C4—C3	−9.0 (2)	C7—C6—C11—C10	0.6 (2)
S1—N2—C4—C5	174.88 (12)	C3—C6—C11—C10	−179.72 (14)
C2—C3—C4—N2	−13.2 (2)	C8—C7—C12—O3	−12.3 (2)
C6—C3—C4—N2	169.49 (14)	C6—C7—C12—O3	167.81 (15)
C2—C3—C4—C5	162.84 (15)	C8—C7—C12—O4	167.35 (15)
C6—C3—C4—C5	−14.5 (2)	C6—C7—C12—O4	−12.6 (2)
C2—C3—C6—C11	104.52 (17)		

### Hydrogen-bond geometry (Å, º)

**Table:**

*D*—H···*A*	*D*—H	H···*A*	*D*···*A*	*D*—H···*A*
N1—H1···O1^i^	0.97 (1)	2.09 (2)	2.9699 (18)	151 (2)
O4—H4···O3^ii^	0.97 (3)	1.64 (3)	2.6103 (19)	177 (3)
O3—H3···O4^ii^	0.97 (3)	1.67 (3)	2.6103 (19)	161 (5)

Symmetry codes: (i) −*x*+2, −*y*, −*z*; (ii) −*x*+1, −*y*+1, −*z*.
